# Cardiovascular consequences of myocardial bridging: A meta-analysis and meta-regression

**DOI:** 10.1038/s41598-017-13958-0

**Published:** 2017-11-07

**Authors:** Sorin Hostiuc, Mugurel Constantin Rusu, Mihaela Hostiuc, Ruxandra Irina Negoi, Ionuț Negoi

**Affiliations:** 1 Department of Legal Medicine and Bioethics, Department 2 Morphological Sciences, Faculty of Medicine, “Carol Davila” University of Medicine and Pharmacy, Bucharest, Romania; 2 Division of Anatomy, Faculty of Dental Medicine, “Carol Davila” University of Medicine and Pharmacy, Bucharest, Romania; 3 MEDCENTER, Centre of Excellence in Laboratory Medicine and Pathology, Bucharest, Romania; 4 Department of Internal Medicine and Gastroenterology, Faculty of Medicine, “Carol Davila” University of Medicine and Pharmacy, Bucharest, Romania; 5 Department of Anatomy, Faculty of Medicine, “Carol Davila” University of Medicine and Pharmacy, Bucharest, Romania; 6 Department of Surgery, Faculty of Medicine, “Carol Davila” University of Medicine and Pharmacy, Bucharest, Romania

## Abstract

Myocardial bridging, a congenital abnormality in which a coronary artery tunnels through the myocardial fibres was usually considered a benign condition. Many studies suggested a potential hemodynamic significance of myocardial bridging and some, usually case reports, implied a possible correlation between it and various cardiovascular pathologies like acute myocardial infarction, ventricular rupture, life-threatening arrhythmias, hypertrophic cardiomyopathy, apical ballooning syndrome or sudden death. The main objective of this article is to evaluate whether myocardial bridging may be associated with significant cardiac effects or if it is strictly a benign anatomical variation. To this purpose, we performed a meta-analysis (performed using the inverse variance heterogeneity model) and meta-regression, on scientific articles selected from three main databases (Scopus, Web of Science, Pubmed). The study included 21 articles. MB was associated with major adverse cardiac events - OR = 1.52 (1.01–2.30), and myocardial ischemia OR = 3.00 (1.02–8.82) but not with acute myocardial infarction, cardiovascular death, ischemia identified using imaging techniques, or positive exercise stress testing. Overall, myocardial bridging may have significant cardiovascular consequences (MACE, myocardial ischemia). More studies are needed to reveal/refute a clear association with MI, sudden death or other cardiovascular pathologies.

## Introduction

Myocardial bridging (MB) is a congenital abnormality characterised by the presence of an intramural course of a coronary artery^1–4^, causing a distinguishing systolic and diastolic flow disturbance^5^. It was usually considered a benign condition, mostly because it constricts the bridged coronary during systole, while most of the blood flow occurs during diastole. Many studies suggested a potential hemodynamic significance of MB^6–16^ and some, usually case reports, implied a possible correlation between MB and various cardiovascular pathologies including acute myocardial infarction^17,18^, ventricular rupture^19^, life-threatening arrhythmias^20,21^, HCM^22–25^, apical ballooning syndrome^26,27^ or sudden death^28^. In forensic autopsies, it is sometimes considered a cause of sudden death, and is often reported as such in scientific articles, especially in association with other congenital cardiac or coronary anomalies^6,22,29–36^. Hong *et al*. published a meta-analysis in which they showed a statistically significant correlation between MB and myocardial infarction (MI), and failed to prove a positive relationship between MB and major adverse cardiac events(MACE)^37^. However, they did not check for potential confounding factors, nor it did perform a detailed analysis of the heterogeneity. Also, since that meta-analysis was published, many more articles about MB were issued in the scientific literature.

The main objective of this article is to evaluate whether MB may cause significant cardiac effects or if it is strictly a benign anatomical variation.

## Materials and Methods

The study was performed by following the PRISMA guidelines for reporting systematic reviews and meta-analyses^38^.

### Selection criteria

Inclusion criteria: (1) studies in which were compared a group of subjects with myocardial bridging and a group without this condition, regarding one of the following endpoints: MACE, cardiovascular (including sudden) death (CVD), MI, myocardial ischemia,; (2) raw data regarding the number of cases from each subgroup (myocardial bridging +/− outcome, no myocardial bridging +/− outcome). We used as exclusion criteria: (1) the absence of relevant information to reconstruct the data needed for analysis; (2) studies not published in English; (3) studies with less than 30 subjects in the cases group; (4) case series/case reports; (5) articles not found in online databases. MACE was defined as composite events of cardiovascular death, myocardial infarction, target vessel revascularization, and stent thrombosis^39^. MI was defined serum cardiac biomarker increases with symptoms of ischemia, ECG changes suggestive for new ischemia/myocardial infarction, or the identification of myocardial infarction at the autopsy. If, in a prospective study were given data about previous MIs at baseline (such as pathological Q waves), they were also included in the analysis. Cardiovascular death was defined as any death with no known non-cardiac underlying causes; we also included in this endpoint sudden deaths and cardiac arrests. Myocardial ischemia was assessed through specific signs as assessed on ECG (ST changes), a positive stress exercise test, or positive Thallium scintigraphy for ischemia.

### Search method

We analyzed the results obtained from three databases: Web of Science, Scopus, and Pubmed, by using the following keywords: “myocardial bridging”, “mural coronary”. We preferred not to use additional, restrictive criteria (e.g. article type) as other varieties (letters, case presentations, reviews) could add relevant data to the meta-analysis (discussions, finding other pertinent studies). The reference list of each appropriate item was inspected for other, potentially relevant studies, to be included in the meta-analysis. The references and abstracts (if available) were imported in Thompson Endnote software, and useful articles were downloaded for further inspection.

### Data collection and analysis

For each study, two reviewers extracted the data separately and included it in Excel Datasheets. We summarised the following information: study, name, the total number of cases in each group, country, general inclusion and exclusion criteria, endpoints, covariates: depth, length, systemic hypertension, angina pectoris, diabetes mellitus, hyperlipidemia, smoking, age, gender.

### Risk of bias

The risk of bias was assessed separately, for each case, by two reviewers. We analysed selection bias (inclusion and exclusion criteria, whether the study was dedicated to MB or had a more general nature), multiple publication biases, measurement bias (the method used), statistical reporting bias. Based on these elements, we separated the studies in three subgroups: high risk of bias, moderate risk of bias and low risk of bias.

### Quality assessment

Quality assessment was performed with the Quality in Prognostic Studies Tool from which we removed study attrition (which was considered not relevant for a prevalence study). For each remaining subscales (study participation, prognostic factor measurement, outcome measurement, study confounding, statistical analysis and reporting), we graded each study as high quality (2 points), moderate quality (1 point) or low quality (0 points) and we summed up the results (Table 1).

**Table 1:** Studies included in the analysis.

Study, Year	Method	Country	Main Inclusion Criteria	Main Exclusion Criteria (Cardiovascular)	Type	Endpoint, Follow-up	Subjects	Quality	Bias
***Brolin***, ***2015*** ^90^	CT, 64	Sweden	Fulfilled the definition of MI Underwent CAG showing no of minimal signs of atherosclerosis	Myocarditis, a clinical diagnosis of PTE, non-sinus rhythm on admission, pacemaker use, patient history of heart disease, obstructive lung disease, renal disease	Case-Control	MI (acute myocardial infarction and non-obstructed coronary artery, screened for the Stockholm Myocardial Infarction with Normal Coronaries study)	54 with MB (28 with MI) 61 without MB (29 MI)	8	Moderate
***Canyigit***, ***2009*** ^91^	CT, 16	Turkey	Suspected CAD, control of coronary stents or bypass grafts	—	Retrospective	Positive effort test (timeframe unspecified)	108 with MB (8 with positive effort test), 162 without MB (13 with positive effort test)	8	Low
***Ishii***, ***1986*** ^58^	Autopsy	Japan	Consecutive autopsy cases	—	Retrospective	MI (location, age not specified)	173 cases with MB (16 MI), 257 cases without MB (20 MI)	8	Low
***Kim Sung Soo***, ***2010*** ^92^	CAG	Korea	Chest pain without significant CAD (defined as <50% stenosis) Discharged with medical therapy and for chest pain	Significant CAD, risk factor causing chest pain (ie, valvular heart disease, cardiomyopathy, congenital heart disease, myocarditis, significant arrhythmia, pulmonary disease, gastrointestinal disease)	Retrospective	MI (diagnosed clinically, no timeframe specified) ECG signs suggestive of myocardial ischemia/infarction, no timeframe specified (ST segment change, pathologic Q wave)	308 with MB (28 with clinically detected MI, 17 with ST segment change, 14 with pathologic Q wave) 376 without MB (15 with clinically detected MI, 10 with ST segment change, 14 with pathologic Q wave)	8	Moderate
***Kim***, ***2007*** ^93^	CAG	Korea		Patients with a 50% stenotic lesion, previous acute coronary syndrome, underlying HCM,	Case-Control	ECG signs suggestive for myocardial ischemia (ST changes) after CAG with Ach provocation test,	81 with MB (34 with ECG changes) 195 without MB (13 with ECG changes)	8	Moderate
***Kitazume***, ***1983*** ^94^	CAG	US	Patients with resting or stimulated intraventricular peak systolic gradients of 20 mm Hg or more (asymmetric septal hypertrophy, idiopathic hypertrophic subaortic stenosis, hypertrophic obstructive cardiomyopathy)	Coexisting CAD, aortic valvular disease, open heart surgery with myomectomy, valve replacement, or bypass grafting	Case-Control	Sudden death, (median follow-up for the control group–68 months, and for the MB group–50 months), pathological Q waves	20 with MB (0 CVDs, 5 with pathological Q waves) 46 without MB (2 CVDs, 6 with pathological Q waves)	6	Moderate
***La Grutta***, ***2012*** ^95^	CT, 64	Italy	Consecutive patients	Known CAD (coronary revascularization by either cardiac surgery or angioplasty)	Retrospective	Positive ECG stress tests	73 with MB (4 with positive stress test) 181 without MB (21 with positive stress test)	6	Low
***Lee***, ***2014*** ^75^	CAG	Korea	Consecutive subjects with implanted Drug Eluting Stents	—	Prospective	MACE (cardiac death, myocardial infarction, TLR, including ischaemic TLR, and stent thrombosis, follow-up–3 years	94 with MB (0 CVD, 3 MI, 21 TLR, 1 stent thrombosis) 457 without MB (10 CVD, 12 MI, 30 TLR, 3 stent thrombosis)	8	Moderate
***Marcos-Alberca***, ***2011*** ^96^	CT, 64	Spain	Stable chest pain and intermediate risk of CAD	—	Prospective	MACE (cardiac death, MI, TLR), follow-up–6.4 months	31 with MB (CVD 0, MI 1, revascularization 2) 43 without MB (CVD 0, MI 1, TLR 0)	6	Low
***Mohiddin***, ***2000*** ^97^	CAG	US	Paediatric subjects with HCM	—	Prospective	Myocardial ischemia as detected by abnormal thallium scintigraphy, sudden death (cardiac arrest). Follow-up up to the age of 20.	23 with MB (17 abnormal exercise thallium scintigraphy, 1 sudden death, 1 cardiac arrest) 34 without MB (14 abnormal thallium cardiac scintigraphy, 1 sudden death, 3 cardiac arrest)	8	Moderate
***Rha***, ***2012*** ^98^	CAG	Korea	Consecutive subjects,	Significant CAD	Prospective	MACE (cardiovascular death, MI, revascularization), unspecified follow-up	367 with MB (0, 0 MI, 2 revascularization) 1027 without MB (2 CVDs, 1 MI, 6 revascularization)	8	Moderate
***Rubinshtein***, ***2013*** ^99^	CT, 64	Israel	Subjects with chest pain syndromes	Obstructive CAD, revascularization	Prospective	MACE (CVD, MI), followed for 6.1 +/− 1 years	117 with MB (4 CVDs, 2 MI) 217 without MB (6 CVDs, 1 MI)	8	Low
***Schwartz***,***2008*** ^100^	CAG	US	Suspected myocardial ischemia, without CAD at coronary angiography	Significant CAD, severe LVH, other cardiac diseases	Case-Control	MI (through ECG changes) SPECT Signs of ischemia, exercise testing	157 with MB (25 MI, 11 ST changes, 51 ischemia/MI through SPECT, 23 + exercise stress testing) 100 without MB (13 MI, 5 ST changes, 36 ischemia through SPECT, 18 + exercise stress testing)	2	Low
***Sheu***, ***2011*** ^101^	CT,64	Taiwan	Subjects that underwent CT for known or suspected coronary artery disease or self-referral for physical check-up	Documented CAD, PTCA, CABG	Prospective	MACE (revascularization), follow-up–21.91 months	89 with MB (0 TLR) 336 without MB (9 TLR)	10	Low
***Sorajja***, ***2003*** ^88^	CAG	US	HCM	absence of any other cardiac/systemic disease, able to cause the observed hypertrophy	Prospective	Cardiovascular death, follow-up - 6.8 years	54 with MB (5 CVDs, of which 0 due to MI) 361 without MB (33 CVDs, of which 3 due to MI)	6	Moderate
***Verhagen***, ***2013*** ^59^	CT, 64	Netherlands	Stable or unstable angina pectoris	PTCA, CABG	Retrospective	History of MI	40 with MB (8 with MI) 88 without MB (21 with MI)	9	Low
***Wang***, ***2008*** ^99^	IVUS	China	Typical or atypical angina	Irregular heart rate, congestive heart failure, chronic pulmonary of kidney diseases	Retrospective	Exercise stress testing	30 with MB (29 with + stress exercise testing) 21 without MB (18 with + stress exercise testing)	7	Low
***Wang***, ***2013*** ^57^	CT, 64	China	Chest pain or suspected CAD	chronic kidney disease, hypertrophic cardiomyopa- thy, valvular or congenital heart disease, and non-sinus rhythm	Prospective	AMI, follow-up 3 years	261 with MB (7 with MI) 2057 without MB (98 with MI)	2	Moderate
***Xiang***, ***2009*** ^103^	CAG	China	Atypical chest pain at rest or after exercise, especially at night	Significant coronary stenosis, heart failure, syncope, Adam-Stokes syndrome, hypertrophic cardiomyopathy, PTCA, valvular disease	Retrospective	Myocardial ischemia on Thallium scintigraphy, + exercise stress testing	68 with MB (45 + for myocardial ischemia using scintigraphy, 48 + for stress exercise testing) 148 without MB (12 + for myocardial ischemia using scintigraphy, 3 + for stress exercise testing)	10	Low
***Yan***, ***2006*** ^104^	CAG	China	Acute STEMI who underwent primary PTCA procedures	Surgery	Prospective	MACE, In Hospital mortality, follow-up up to 6 months	46 with MB (12 CVDs, 2 TLRs) 508 without MB (29 CVDs, 18 TLRs)	1	High
***Yetman***, ***1998*** ^28^	CAG	Canada	Pediatric subjects with HCM	Other potential causes for cardiac hypertrophy	Prospective	ECG changes (ST-segment changes at initial admission), cardiac arrest, death. Follow-up 7.1 years	10 with MB (3 sudden death, 4 cardiac arrest, 7 ST changes) 26 without MB (2 sudden death, 0 cardiac arrest, 9 ST changes)	8	High

### Statistical analysis

We determined the effect size using inverse variance heterogeneity model computed in Microsoft Excel 2016 with the MetaXL add-on version 5.3 and used Comprehensive Meta-Analysis v3 for performing meta-regression. For each group and subgroup, we performed a forest plot. For the analysis of publication bias, we used the funnel plot and the Luis Furuya-Kanamori (LFK) index. We used a continuity correction of 0.5, and 95% confidence intervals. Forest plots were done using Microsoft Excel 2016 with the MetaXL add-on 5.3, with the effect size transformed logarithmically for better viewing.

## Results

### Search synthesis

**Figure 1:**
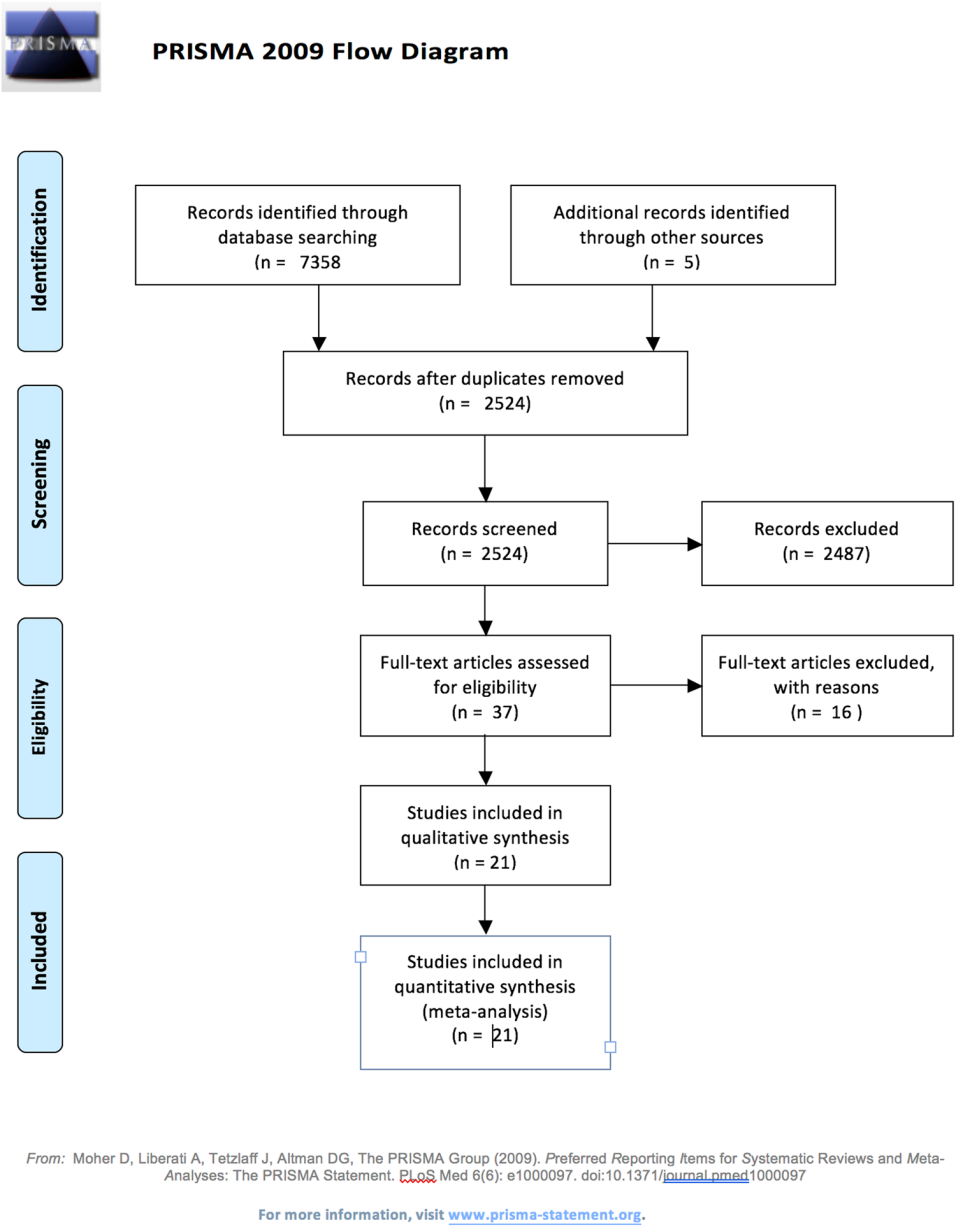
PRISMA flow diagram (The PRISMA Statement and the PRISMA Explanation and Elaboration document are distributed under the terms of the Creative Commons Attribution License, which permits unrestricted use, distribution, and reproduction in any medium, provided the original author and source are credited)^40^.

During the initial database research, we obtained 7358 articles from which, after deleting 4839 duplicates, we were left with 2519, that were included in the initial analysis. From them, we screened the abstracts and removed all articles not containing clinical studies that included a group of subjects with myocardial bridging and a group without this condition, regarding the above-mentioned endpoints. 32 articles selected and further downloaded. By analysing their references, we found another 5 potentially relevant items, which were also downloaded. The next step included the analysis of the full text manuscripts, after which we selected 21 to be included in the meta-analysis, which fully respected the inclusion criteria. Details about search synthesis are presented in Fig. 1 ^40^. We detailed the papers comprised in the analysis in Table 1.

### Major Adverse Coronary Events (MACE)

**Figure 2:**
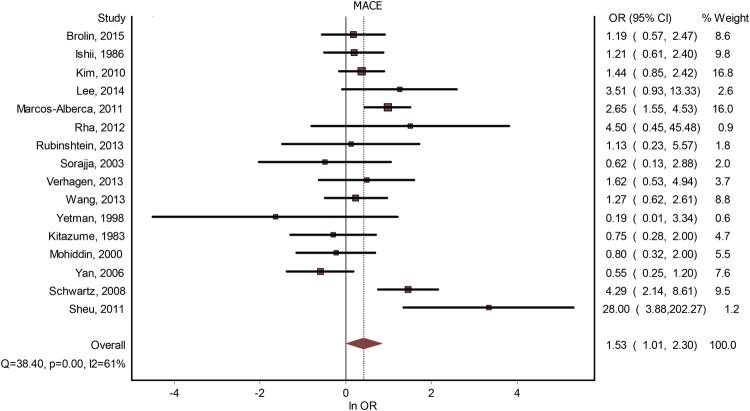
MB and MACE. Forest plot.

**Figure 3:**
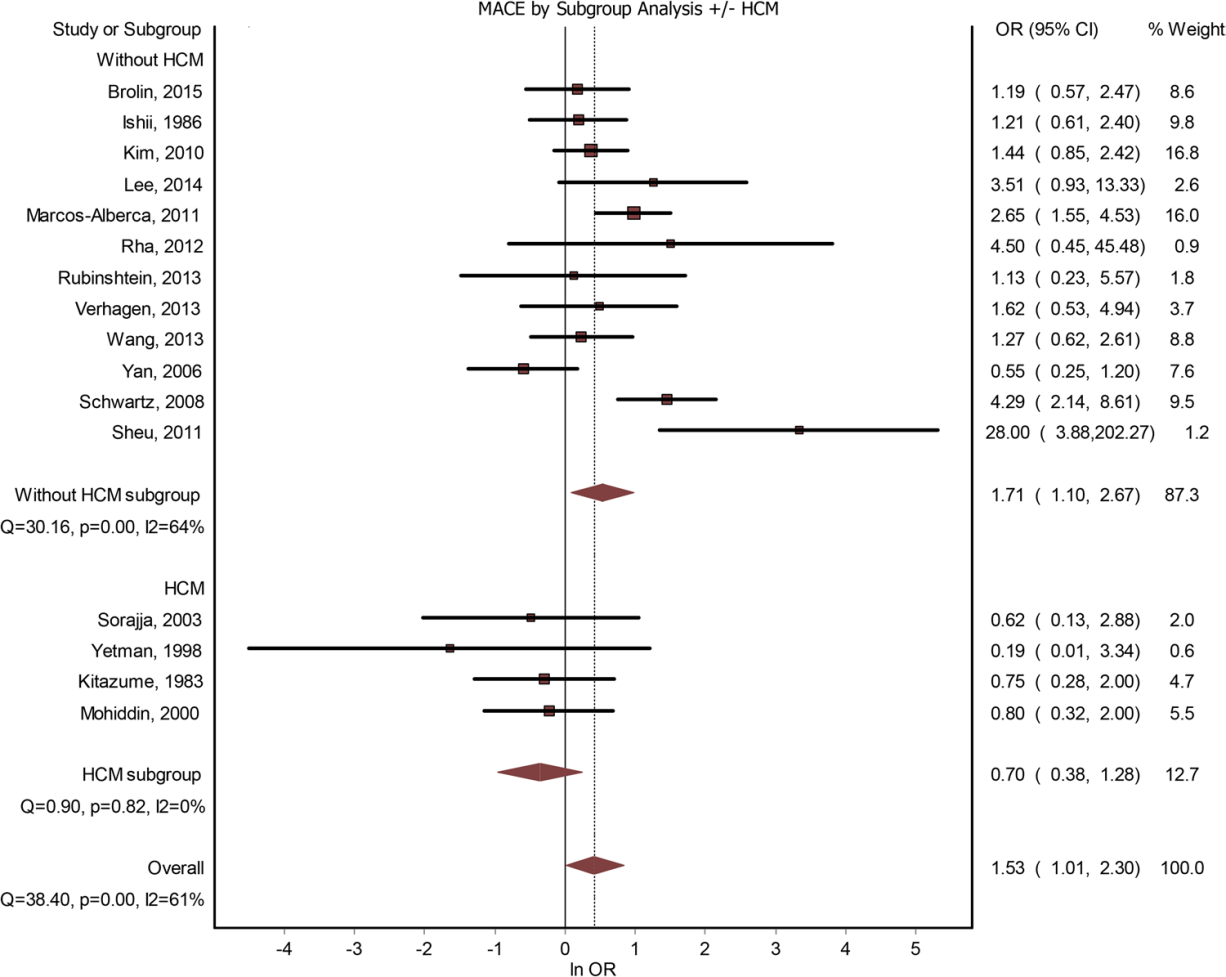
Subgroup analysis. MACE with/without HCM. Forest plot.

Sixteen studies contained data about a potential correlation between MB and MACE. The presence of MB led to a significantly increased risk for MACE - OR = 1.53(1.01–2.30), p < 0.001 (Fig. 2). The publication bias was minimal, with an LFK index of 0.33. The heterogeneity of the included studies was moderate (I^2^ = 61%). Meta-regression analysis found a positive correlation between MACE risk and the increase in age differential between subjects with MB versus subjects without MB; gender, systemic hypertension, hyperlipidemia, previous angina, diabetes mellitus or smoking failed to generate statistically significant differences (see Table 2). By performing a subgroup analysis, based on whether the studies considered significant coronary artery disease as an either inclusion or exclusion criteria, we found that, if the presence of CAD was used as an inclusion criteria, OR was 1.60(0.79–3.23), and if it was used as an exclusion criteria – 1.54 (0.79–3). By performing a subgroup analysis, based on whether the studies were done on patients with HCM, we found that studies that were not done specifically for HCM had an OR = 1.71 (1.10–2.67), while studies that were done on subjects with HCM had an OR = 0.60(0.38–1.28). See Fig. 3.

**Table 2:** Z values for meta-regression analysis.

Effect	Age	Gender	HTA	Hyperlipidemia	Diabetes	Angina	Smoking	Depth	Length
***MACE***	**2**.**71 (p** = **0**.**068)**	−0.12	1.14	−0.23	0.68	1.02	−0.82	0.66	1.56
***AMI***	0.16	0.36	−0.19	−0.29	0.53	0.02	0.53		
***CVD***	2.39 (p = 0.017)	−0.09		0.24	—	—	—		
***Myocardial ischemia***	0.09	−0.94	−0.91	−0.75	0.23	0.16	1.15

### Myocardial infarction

**Figure 4:**
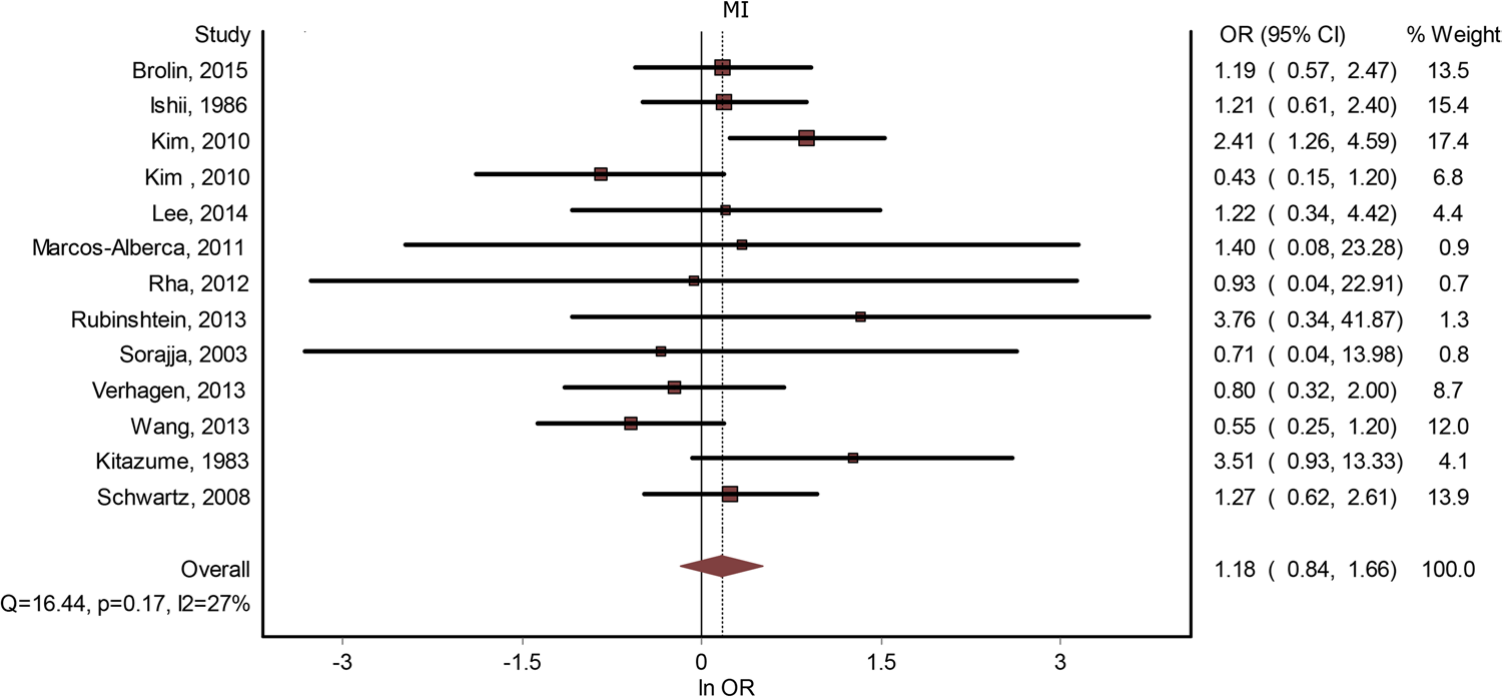
MB and MI. Forest plot.

Thirteen studies contained data about a potential correlation between MB and MI. The presence of MB did not lead to a significantly increased risk for MI - OR = 1.18(0.84–1.66), p = 0.30 (Fig. 4). The publication bias was minimal (LFK index = 0.30). The heterogeneity of the included studies was low (I^2^ = 27%). Meta-regression analysis did not show any significant effects on the result determined by age, gender, systemic hypertension, hyperlipidemia, previous angina, diabetes mellitus or smoking (see Table 2). By performing a subgroup analysis, based on whether the studies considered significant coronary artery disease as an either inclusion or exclusion criteria, we found that, if the presence of CAD was used as an inclusion criteria, OR for MI was 0.74(0.43–1.25), and if it was used as an exclusion criteria – 1.45 (0.87–2.39).

### Cardiovascular death

**Figure 5:**
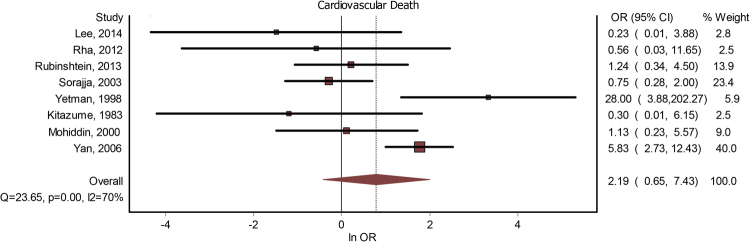
MB and CVD. Forrest plot.

Eight studies included data about a potential correlation between MB and cardiovascular death. The presence of MB did not lead to a significant increase in the risk of cardiovascular death - OR = 2.19(0.65–7.43) (Fig. 5). The publication bias is minor (LFK index = −1.28). The heterogeneity of the included studies was moderate to high (I^2^ = 70%). Meta-regression analysis found a positive correlation between CVD risk and the increase in age differential between subjects with MB versus subjects without MB; (see Table 2).

### Myocardial ischemia

**Figure 6:**
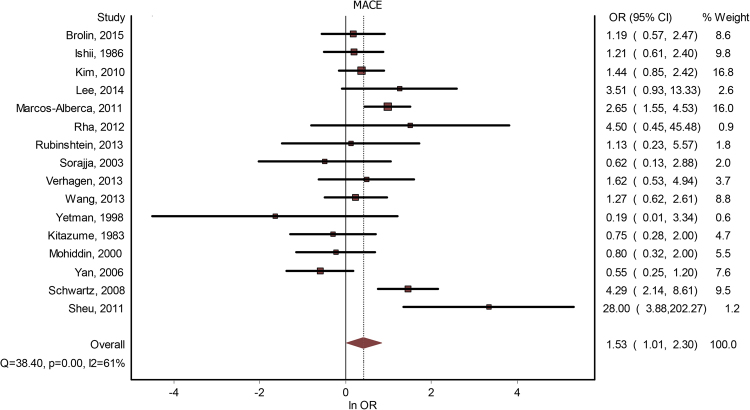
Myocardial ischemia and MB. Forest plot.

Nine studies included data about a potential correlation between MB and myocardial ischemia. Two studies contained more than one method of assessing myocardial ischemia (Xiang and Schwartz), and we took into account in both cases the outcome with the most number of positive subjects. The presence of MB was significantly associated with myocardial ischemia - OR = 3.00(1.02–8.82) (Fig. 6). The publication bias was minor (LFK index = −1.68). The heterogeneity of the included studies was high (I^2^ = 89%). Meta-regression analysis did not show any significant effects on the result determined by age, gender, systemic hypertension, hyperlipidemia, previous angina, diabetes mellitus or smoking (see Table 2).

## Discussions

Our study revealed that, overall, MB increases the risk for MACE and myocardial ischemia, and subsequently a possible connection between it and various cardiovascular pathologies cannot be excluded. The physiological characteristics and morphological consequences of MB are still not yet fully elucidated, even if there are many studies analysing specifically with these issues.

Even if MB mainly causes a systolic narrowing of the affected coronary, both cardiac phases are affected. Intracoronary Doppler showed a finger-tip-like anterograde flow of the blood in most MB patients, characterised by a sharp increase in coronary flow velocity during early diastole, followed by a steep decrease in speed, leading to a plateau in mid-late diastole^41^. During systole, the anterograde flow is decreased, absent, or even reversed, due to the systolic squeezing of the bridged coronary segment^41^. Ge *et al*. showed the pressure within the segment proximal of the MB to be much higher than the pressure in the aorta and that the maximum pressure in the proximal segment is reached in mid-systole compared to normal segments is reached in the end-systole^42^. During end-diastole, the myocardium dilates, triggering a negative pressure in the mural segment, and an increased pressure gradient between the bridged segment and the neighbouring areas(the so-called sucking phenomenon)^42^. The systolic compression is usually eccentric, and only rarely concentric, leading to a half-moon like echo-lucent area neighbouring the bridge when viewed through IVUS^41^. Previous studies showed that systolic compression by the bridge could be either elliptical, associated with a 50% reduction of the luminal area, or concentric, associated with a 75% reduction^43^. Other authors showed that, if superficial and short MBs do not lead to significant hemodynamic effects, this is not the case with deep and long variants, which may cause severe ischemia or cardiovascular events^44^. Hostiuc *et al*. showed that hemodynamically significant MB is associated with increased fibrosis and interstitial edema in subjects who died suddenly and had no other apparent cause of death, suggesting that the most likely cause of death was electrical instability^6^, which is known to be associated with increased myocardial fibrosis^45,46^.

Endothelial dysfunction is an early phase of atherogenesis^47^. Numerous studies reported an alteration of the normal endothelial physiology associated with MB, including an abnormal response to acetylcholine (ACh) or decreased responses to NO and endothelin-1. Acetylcholine acts as an endothelium-dependent vasodilator, by stimulating the release of EDRF, if the endothelium is intact^48^. If however there are endothelial lesions (including atherosclerotic disease), ACh will cause smooth muscle constriction^49^. ACh-generated vasoconstriction was often showed to be associated with MB. For example, Kuhn revealed that vasoconstriction after the administration of ACh is higher in the coronary segment containing a MB compared to non-bridged segments^50^. Kim *et al*. showed that, after infusion with ACh, subjects with MB have a statistically significant increase in focal vasoconstriction in the mural segment, compared to similar, non-bridged coronary segments from the control group^51^. Teragawa showed that subjects with MB have an almost two-fold increase in the rate of coronary spasm compared with the control group^52^. Zoghi *et al*. showed, by measuring flow-mediated dilatation in the brachial artery, that the presence of MB also leads to remote vasoconstrictive effects^53^. Herrmann demonstrated that MB causes an increased shear stress and significant vasoconstriction after the administration of ACh without changes in the coronary blood flow^16^. Masuda *et al*. found that bridged LAD segments have a weaker immunoreaction to endothelial nitric oxide synthase (eNOS) and endothelin-1(ET-1) compared to the proximal and distal segments^54^. NO leads to vasodilatation, and it might be involved in the “sucking phenomenon” described by Ge^42^.

The segment proximal to the MB is put at a high wall shear stress due to the significant pressure gradients between the bridged area and the proximal coronary segment^42^, potentially causing a chronic coronary pressure overload (CCPO)^16^. CCPO is associated with significant endothelial changes, including intimal thickening, medial thickening, disruption of the internal elastic lamina or endothelium^55^, favouring the appearance of atherosclerosis.

Nakaura *et al*. showed that mid-LAD MB is strongly positively associated with atherosclerosis (OR = 4.99). They did not mention, however, the location of the atherosclerotic plaques (distally, intramural or proximal from the bridged area). Hong *et al*. found that the prevalence of lesions located distally from the MB was significantly lower compared to those located proximally (5.9% versus 62.4%). In their article age, male gender, a history of diabetes mellitus, systemic hypertension, bifurcated vascular lesions, smoking index, or hyperlipidemia increased the severity of the coronary stenosis^56^. Wang *et al*. showed that MB (both superficial and deep) is associated with non-significant stenosis proximal to the bridged area and that MB in middle LAD is positively associated with stenosis in non-bridged arteries. The most likely explanation for the latter is the fact that deep MB has hemodynamic significance, causing a disturbed flow in the other coronaries, and therefore increases the frequency of atherosclerotic deposits^57^. By compressing the tunnelled artery during systole, MB increases lymph drainage of the vessel wall, protecting against lipid accumulation within the bridged coronary wall^57,58^. Verhagen *et al*. suggested that the reason for the lack of atherosclerosis under the bridged coronary is represented by a lack of exposure to perivascular adipose tissue at that site, which is known to secrete pro-inflammatory adipokines and cytokines^59^. Saidi *et al*., on the other hand, showed that between the bridged muscle and the coronary is sometimes interposed an adipose layer, which they hypothesised to act as a cushion, limiting the compressing forces acting upon the bridged coronary segment^60^. However, this layer would, in fact, secrete pro-inflammatory adipokines and cytokines. Locally increased eNOS is known to be associated with increased atherosclerosis in coronary arteries^54,61^; therefore, increased eNOS levels proximally from the bridged segment might be involved in the development of proximal segment atherosclerosis. Another explanation for the distribution of atherosclerosis/around the bridged area might be represented by the shape of the endothelial cells, which are spindle-shaped under the bridge, suggesting they are under a high shear stress, while in other regions they have a polygonal distribution, suggesting they are subjected to a decreased shear stress^58,62^. Increased shear stress seems to decrease monocyte adhesion and lipid transfer across the vascular wall, therefore decreasing the risk for atherosclerosis^59,62^.

Bilen *et al*. showed that subjects with MB have a significantly increased mean platelet volume (MPV). Larger platelets are associated with increased reactivity and production of more prothrombotic factors^63^. Potential causes for this result might be a reduced production of prostacyclin and nitric oxide, impairment of endothelial-dependent vasorelaxation, increased proximal atherosclerosis, increased shear stress^64^. This increased thrombogenic status might favour the development of coronary thrombosis or might increase its severity.

Numerous studies suggested or proved that MB is associated with various types of physiological changes. Ge *et al*. showed that MB leads to a decreased coronary flow velocity reserve, which remains higher proximally compared to distally from the bridged segment; also, coronary flow velocity reserve seems to be more affected in subjects with severe coronary stenosis^41^. Schwartz *et al*. quantified the decrease in the coronary flow reserve caused by MB – they found a mean ratio of 2.0 distally from the bridged coronary artery (normally the value is above 3)^7^. Fractional flow reserve is often used to quantify the physiological significance of CAD. It is not influenced by heart rate or blood pressure changes^65^, nor by dobutamine challenge in subjects with fixed CAD^66^. Diefenbach *et al*. showed that inotropic challenge leads to a significant increase in the degree and length of MB segments, as evaluated on CAG, compared to those obtained at rest in symptomatic subjects, suggesting the presence of a specific coronary physiological response to inotropic stimuli^67^. Yoshino *et al*. proved that fractional flow reserve (FFR) to dobutamine challenge is significantly decreased in the presence of microvascular endothelial dysfunction^65^. Heart rate recovery is a prognostic tool for the cardiac autonomic functions^68^; increased heart rate recovery (HHR) was associated with decreased parasympathetic activity^69^, a higher susceptibility for atherosclerosis, and increased five-year mortality^70^. Okutucu showed the presence of MB on LAD to be a significant predictor for increased HHR at 1 minute (b = −8.524; 95% CI −14.934 to −2.113; p = 0.009), and argued that the finding was caused by silent or symptomatic myocardial ischemia. QT dispersion (QTd), defined as the minimum QT interval subtracted from the maximum, is an effective marker for electrical myocardial heterogeneity; higher values are associated with an increased risk of cardiac arrhythmias^71^. Aksan *et al*. demonstrated that subjects with MB have a significantly increased QTd during peak exercise compared with its baseline value (42.6 ms versus 36.4 ms), while in non-MB subjects the increase during peak exercise was not statistically significant (29.1 ms, versus a baseline value of 27.1 ms)^72^.

All these results come to confirm that, at least in some cases, MB may have various cardiovascular consequences. Our study showed that MB presence is associated with an increased risk of MACE and myocardial ischemia, but failed to show a possible positive correlation with CMH, MI, or cardiovascular death. However, even if these associations did not reach statistical significance, the variability of the confidence intervals is very high, which can be caused either by the heterogeneity of the studies included in the meta-analysis, or by a highly variable response to this condition (possibly dependent upon length, depth or the MB, its location, associated pathologies, and so on). The scientific literature analysis showed or suggested a series of associations between MB and cardiovascular pathologies, including, coronary spasm, MI, HCM, Takotsubo cardiomyopathy, arrhythmias, or sudden death.

The association between coronary artery spasm and MB was first implied by Grover *et al*., who reported the case of a patient who developed CAS during cardiac pacing, an association that was later on confirmed by other studies (see e.g. ref.^73^). Recent articles showed that patients with MB have a higher incidence of coronary vasospasm induced by ACh. Sung *et al*. showed that patients with MB who responded to lower doses of ACh had a more severe decrease of the luminal diameter, and had a worse cumulative clinical outcome after 12 months compared to subjects who needed larger ACh doses for a positive response. The mechanical stress caused by the systolic narrowing is known to lead to endothelial dysfunction. Subjects with variant angina are known to be hyperactive to vasoconstrictor stimuli^74^. Atherosclerotic epicardial vessels may present a paradoxical vasoconstriction caused by a direct stimulation of the mural smooth muscle, as a result of widespread endothelial dysfunction^49^. Corroborated, these results might suggest that the coronary vasospasm associated with MB might lead to the development of myocardial ischemia, especially in the presence of other coronary pathologies^71,75^.

De Agustin *et al*. showed that subjects with chest pain and without severe coronary artery stenosis are significantly more likely to have MB compared to those with severe CAD; moreover, in the MB subgroup hyperlipidemia was considerably less often found compared to the group of subjects without MB^76^. Tang *et al*. showed that, in subjects with MB, cardiac ischemia is strongly associated with the degree of mean systolic narrowing, and suggested that the degree of systolic occlusion by the MB is responsible for the appearance of myocardial ischemia^77^. Angelini *et al*. analysed the presence of MB in subjects with and without coronary artery disease, finding that its incidence is significantly lower in the group without CAD^43^. They also showed that there is a higher incidence of MB in men and in subjects with a systolic overload of the left ventricle.

There are two core mechanisms through which MB is thought to cause myocardial ischemia and MI - the development of atherosclerosis proximally from the bridged segment^78^, and a direct compression of the coronary artery by the MB^79^. In subjects with MI, the thickness of the MB is, on average, greater compared to the ones found in subjects without MI^80^. Takamura *et al*. studied two groups of subjects: one with MI in the area supplied by the LAD and without MB on LAD and one with MI in any myocardial area associated with MB on LAD, and found that, if the acute occlusion occurred in the LAD segment proximal from the MB, the length and thickness of the MB were significantly greater, and the distance from the ostium of the LCA to the MB was significantly shorter compared to those without an acute occlusion on the LAD proximally to the MB^81^.

The blood flow during systole is small compared to the one from diastole in normal conditions; this is not the case in tachycardia, when the end-diastolic volume decrease is greater compared to end-systolic volume^82^, potentially leading to myocardial ischemia. In this instance, the blood supply of the myocardium becomes more dependent upon the systolic blood flow, which is impaired by the bridge. There were some cases published in the scientific literature in which tachycardia was associated with MB. Feld, for example, described a patient who, during stage 5 of the Bruce protocol, developed significant flat ST depressions, followed by a complex tachycardia with a rate of 280 bpm, associated with a MB on LAD^20^. Den Dulk *et al*. presented the case of a patient with exercise-induced paroxysmal atrioventricular block^21^. Kracoff *et al*. published the case of a 35 years-old patient, with recent onset angina, who developed episodes of syncope caused by ventricular tachycardia, which was suggested to have been caused by a myocardial bridge^83^. Left ventricular systolic dyssynchrony or intraventricular dyssynchrony, is characterized by a delay or heterogeneity in the timing of contraction in different myocardial segments of the left ventricle. It is usually found in heart failure, and it is proven to cause a more severe myocardial disease and a poorer cardiovascular prognosis. Cai Wei *et al*. showed that mid-LAD MB is associated with an increased prevalence of left ventricular systolic dyssynchrony (LVSD) compared to subjects without MB; moreover, the stenosis caused by the MB and the length of the mural segment have a synergistic effect on the development of LVSD^84^.

Lee *et al*. found that MB is an independent predictor for MACE in patients with eluting drug stents (adjusted OR = 2.897)^75^. Similarly, Tsujita *et al*. found that subjects undergoing eluting drug stents for coronary artery disease in the proximal part of the LAD, and who had detectable MB distal of the stented lesion, had a significantly decreased minim stent area at the end of the procedure, and a higher target lesion vascularization rate^85^.

Hypertrophic cardiomyopathy, an inherited cardiovascular disease that can lead to SCD, heart failure, or stroke, is believed to be associated very often with MB^86,87^. Earlier studies implied a possible correlation between HCM with MB and SCD in some subgroups of patients. For example, Yetman *et al*. showed that MB in children with HCM is a risk factor for SCD^28^. However, more recent ones failed to prove a positive correlation. Tian *et al*. showed that, in autopsy cases, MB is not associated with SCD in HCM^44^. Basso *et al*. found that patients with HCM who died suddenly do not have a higher prevalence of MB compared to subjects with HCM who died from heart failure or other modes of death; from the analysis of their results, however, the age of the subjects with SCD was much lower (22 years, compared with 54 and 67 in the other two groups), the length and depth were higher, and the proportion of associated CAD much lower^87^. Sorajja *et al*. showed that there is no statistically significant difference regarding five-year survival between subjects with and without MB^88^. Our study showed that, when we excluded studies in which HCM was used as an inclusion criteria, MB increased the risk for MACE. More studies are however needed in the area before either establishing or excluding a causal link between these two pathologies.

Some studies suggested that MB is more frequent in patients with apical ballooning syndrome^26,89^, but not in midventricular or basal ballooning^89^; in these cases, MB might influence the development of Takotsubo cardiomyopathy through the generation of microvascular dysfunction and altered sympathetic activity, possibly associated with recurrent LAD segments^89^; more studies are needed in this area to reach definite conclusions.

### Limits

A very high variability of the results, associated with a limited number of studies for testing associations between MB and various cardiovascular consequences. Many studies did not include comprehensive inclusion and exclusion criteria, or complete descriptive data, limiting the number of subgroup analyses we were able to perform. Due to the presence of highly different methodologies, and inclusion and exclusion criteria, there can be a significant selection bias, which was attempted to be minimized through subgroup analyses and meta-regression.

## Conclusions

Overall, myocardial bridging may have significant cardiovascular consequences (MACE, myocardial ischemia). More studies are needed to reveal/refute a clear association with MI, sudden death or other cardiovascular pathologies.
